# Supplementary figures and images for: Proteomic Profiling of Thigh Meat at Different Ages of Chicken for Meat Quality and Development
Source: Foods. 2023 Jul 30;12(15):2901. doi: 10.3390/foods12152901 (PMC10418907; doi:10.3390/foods12152901)

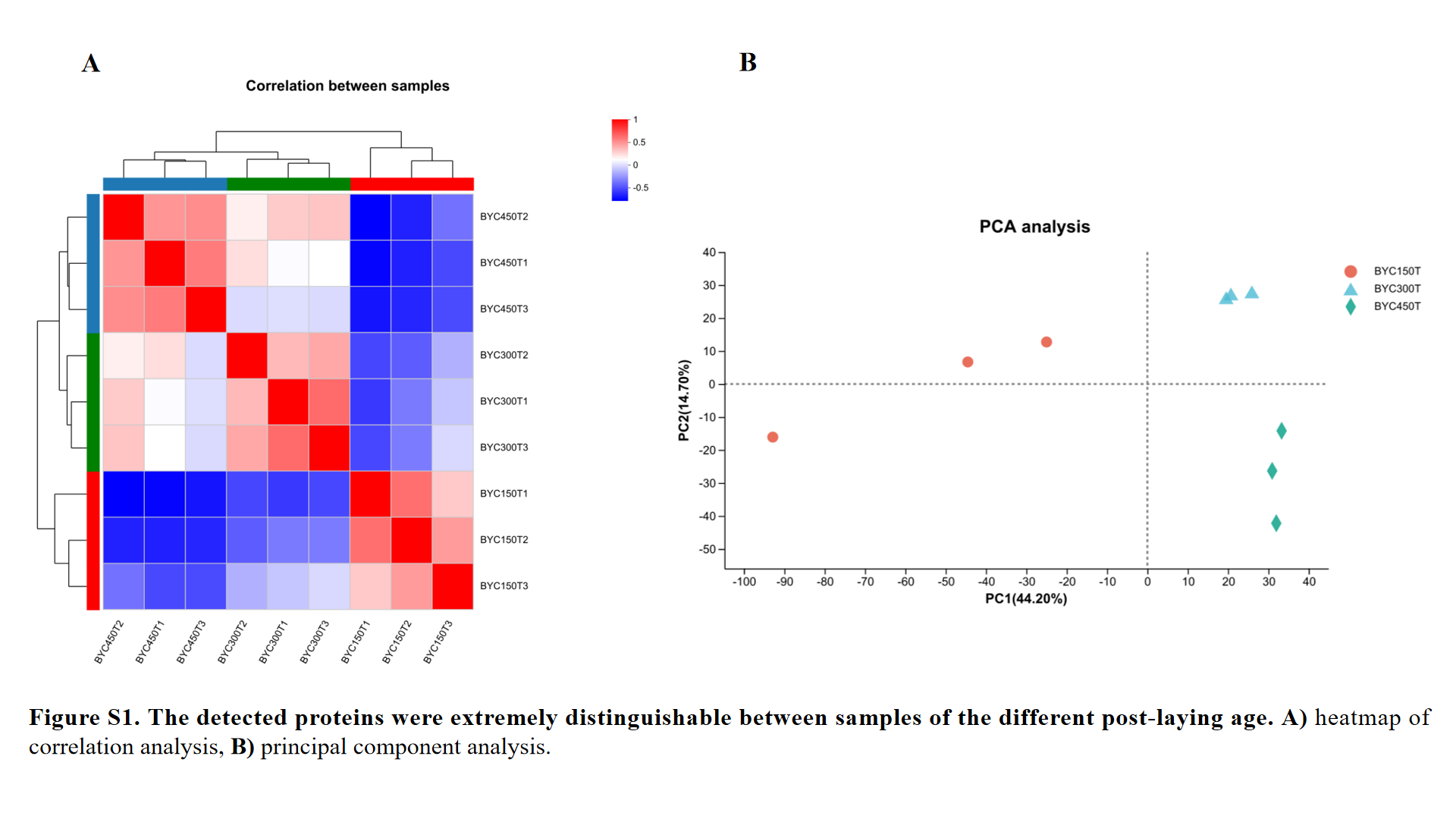

Supplement: Supplementary file 1 [file foods-12-02901-s001.zip › figure supplementary/Figure S1 The detected proteins were extremely distinguishable between samples of the different post-laying age.png]

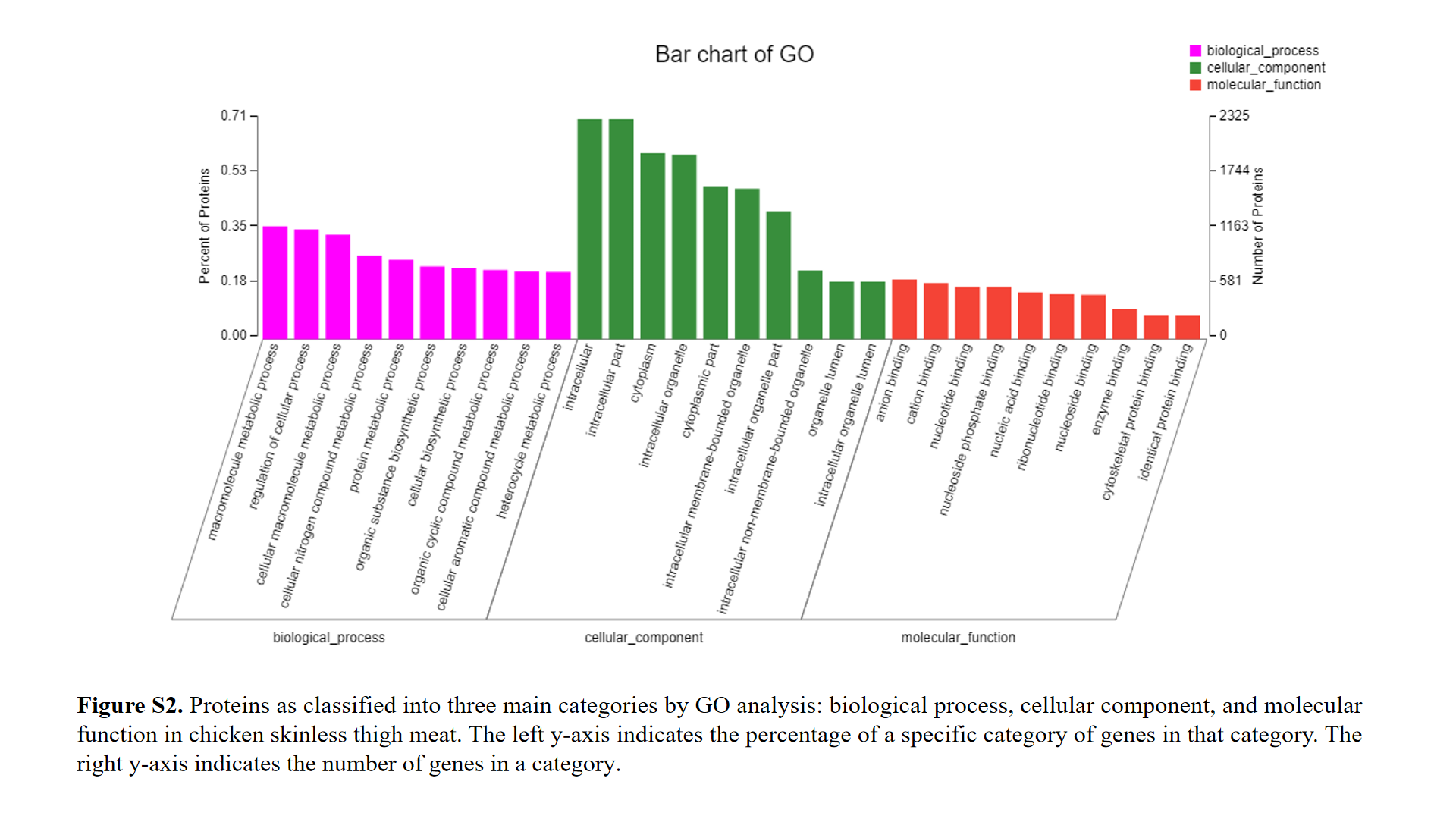

Supplement: Supplementary file 1 [file foods-12-02901-s001.zip › figure supplementary/Figure S2 GO annotations analysis for all detected proteins.tif]

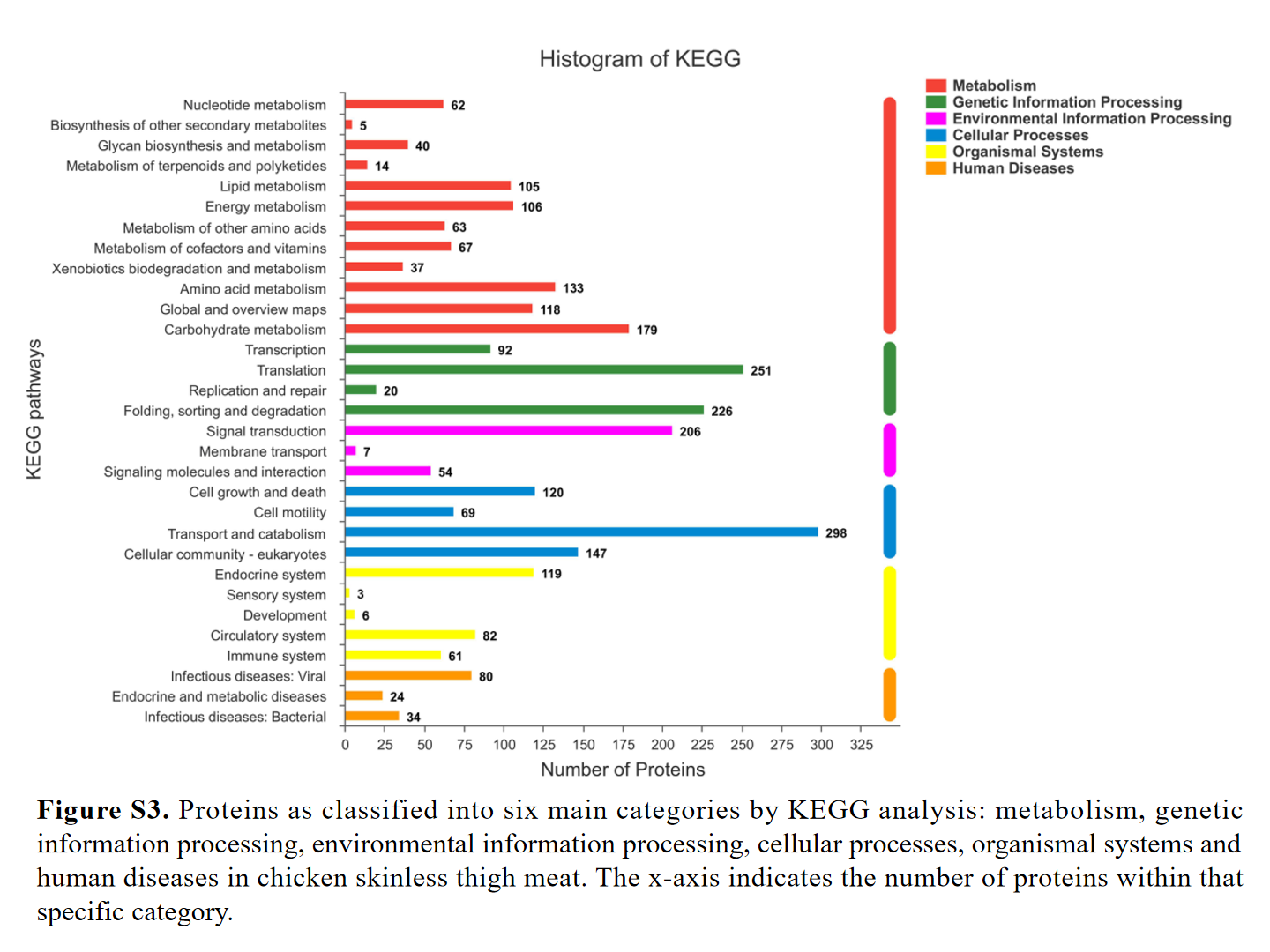

Supplement: Supplementary file 1 [file foods-12-02901-s001.zip › figure supplementary/Figure S3 KEGG annotations analysis for all detected proteins.tif]

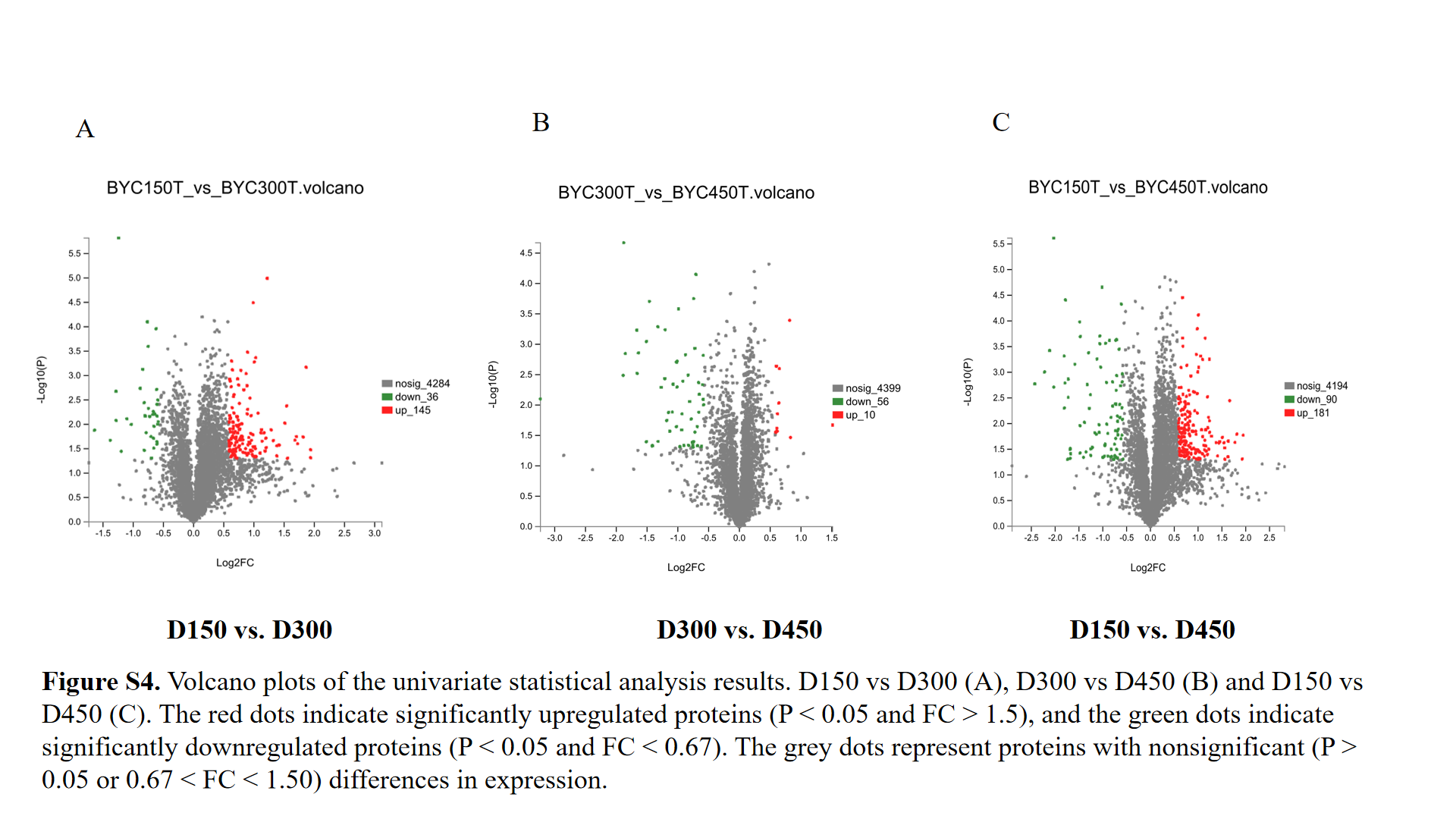

Supplement: Supplementary file 1 [file foods-12-02901-s001.zip › figure supplementary/Figure S4 Volcano plots of the univariate statistical analysis results.tif]

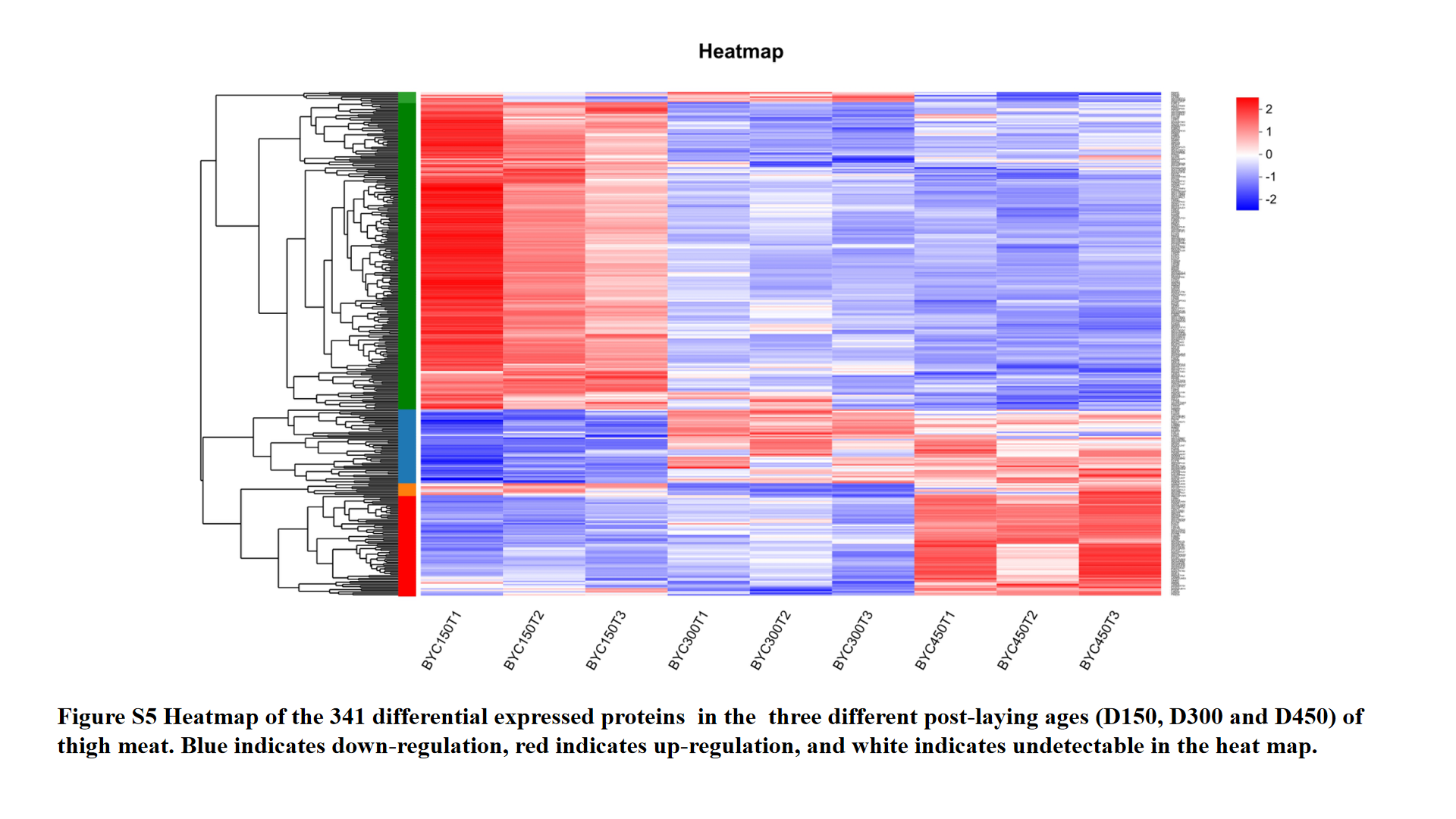

Supplement: Supplementary file 1 [file foods-12-02901-s001.zip › figure supplementary/Figure S5 Heatmap of the 341 differential expressed proteins in the three different post-laying ages (D150, D300 and D450) of thigh meat.tif]

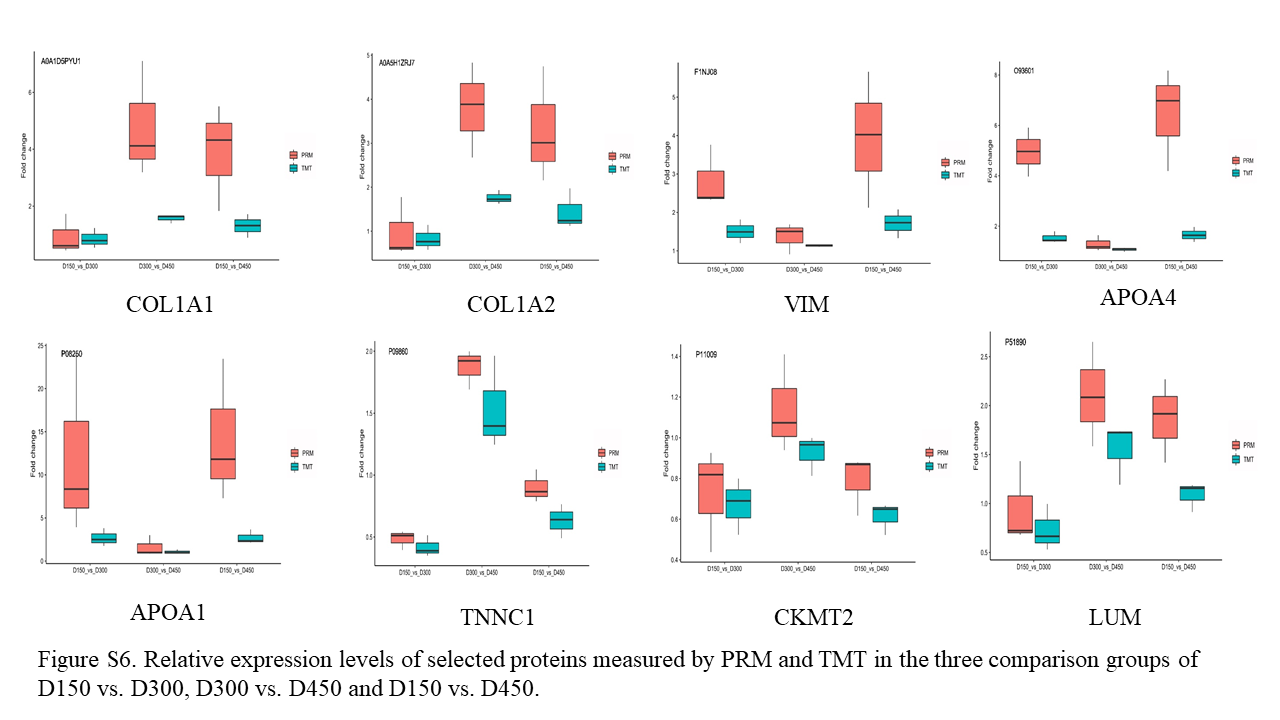

Supplement: Supplementary file 1 [file foods-12-02901-s001.zip › figure supplementary/Figure S6 Relative expression levels of selected proteins measured by PRM and TMT in the three comparison groups.tif]
